# Scaling Up the Evidence: What Open Banking Brings to Gambling Research and Policy

**DOI:** 10.1007/s10899-025-10438-3

**Published:** 2025-10-13

**Authors:** Steven Murphy, Naomi Muggleton

**Affiliations:** 1 Behavioural Science Group, Warwick Business School, Coventry, UK; 2 Gambling Commission, Birmingham, UK

The study ‘Across the Bettor-Verse: An Open Banking Perspective on Gambling in the United Kingdom’ (Ghaharian et al., 2025) represents a timely and innovative contribution. It draws on the UK’s unique Open Banking laws to study gambling in everyday financial behaviour. By analysing individuals’ transaction records, it moves beyond self-report surveys and operator data to provide a more comprehensive, objective view. This study sits alongside a growing set of analyses using transaction data to advance understanding of gambling and harm (Muggleton et al., 2021; Ghaharian et al., 2023; Marionneau and Nikkinen, 2024; Zendle and Newall, 2024; Lahtinen et al., 2025). This methodological shift marks a promising new frontier for gambling research (Volberg, year; Muggleton, 2024), with the potential to yield insights not accessible through conventional methods.

Exploiting individuals’ financial records at scale reflects broader shifts in the field over the past decade, away from a narrow focus on ‘problem gambling’ as a minority issue towards a public health perspective. This approach acknowledges that gambling can generate population-level harms not addressable by targeted interventions alone. The shift bears strong parallels with public health strategies on sugar, alcohol, smoking, and obesity (Marmot, 2005; Rehm et al., 2009; Moodie et al., 2013; Pechey and Marteau, 2018). More recently, scholars have emphasised the need to confront industry influence and frame gambling as a public health issue (Van Schalkwyk et al., 2021; Price, 2021; Wardle et al., 2024; Ukhova et al., 2024). Despite these calls, much of the empirical literature still relies on cross-sectional surveys or small clinical samples, limiting assessment of harms across the wider public (Wardle et al., 2019; Abbott, 2017). Open Banking data therefore represents a key opportunity to align gambling research with modern public health approaches. By enabling objective, large-scale measurement of financial transactions, it provides a rare opportunity to examine gambling participation, expenditure, and financial consequences across the population. Unlike self-report surveys prone to bias, or operator data restricted to single platforms, banking transactions capture gambling as part of household budgets and economic routines. This capacity to situate gambling within the broader financial lives of individuals makes Open Banking data particularly suited to assessing prevalence and harm across the population. Furthermore, unlike most survey-based evidence sources, financial transaction data is inherently longitudinal, allowing monitoring over time and potentially opening the door to causal analyses.

Population-level analyses grounded in objective financial data support upstream, preventative approaches to gambling harm. A major strength of Ghaharian et al. (2025) is its scale. The authors analyse transactions from over 1,030,000 UK individuals, far larger than most prior studies (Lahtinen et al., 2025; Zendle and Newall, 2024). Such breadth offers statistical power and captures behavioural diversity across demographics and platforms. Crucially, the data span multiple gambling operators per person, overcoming the “silo” problem of single-operator studies and offering a holistic view of gamblers’ activity. This comprehensive coverage is critical for accurately identifying risky behaviour: high-spending gamblers often use multiple operators, and only a multi-operator dataset can capture this. By using bank records, the study objectively captures spending participants might forget or misreport, improving accuracy over self-reports.

Another notable strength of this study is its use of Open Banking as the data source. This approach provides access to transaction data across institutions and operators, with participant consent. Crucially, it avoids the practical and ethical challenges associated with alternative sources. For instance, while obtaining a large random sample from a retail bank ensures representativeness and full coverage, it is often prohibitively difficult due to institutional risk aversion. Conversely, data from a gambling operator offer detailed behavioural data but raise concerns about perceived researcher independence and operator influence on research findings and, by extension, the wider evidence base.

The authors note a discrepancy between participation levels in their dataset and official statistics (Gambling Commission, 2022), as their estimates are higher. They suggest several possible explanations, including methodological differences and the demographic composition of the sample. Understanding these differences is an important area for future research and could complement ongoing efforts to improve the quality of official statistics (Office for Statistics Regulation, 2025). Additionally, examining demographically representative subsets of large Open Banking datasets could clarify divergences from national benchmarks.

Another potential source of bias, noted by the authors, relates to the financial circumstances of participants: the dataset includes only individuals applying for credit ratings. In common with other financial transaction datasets, it also excludes cash gambling, which remains a significant component of the market in Great Britain. Given evidence that individuals experiencing gambling disorder often take steps to conceal their behaviour, including through cash gambling (Gambling Commission and NatCen, 2025), there is a risk that gambling by those most vulnerable to harm is under-represented. Many of these limitations, though varying in degree, are common across such datasets. However, the comprehensive descriptive statistics provided by the authors serve as a valuable benchmark for adjusting for bias in future research and policy analysis.

The implications of this research extend beyond academia. It offers a critical resource for regulators and policymakers at a time when many jurisdictions are reducing legal and regulatory barriers to gambling (Harris, 2020; Aimo et al., 2023). As markets liberalise and new operators enter, understanding participation, behaviour, and harms becomes increasingly challenging, yet no less essential for effective regulation. Traditional research methodologies (Sturgis, 2024) face inherent limitations, prompting a shift towards drawing on diverse data sources (Gambling Commission, 2025). Open Banking is already applied to policy contexts (Gambling Commission, 2024). High-quality academic research such as this ensures analysts and decision-makers understand the strengths and limitations of these datasets when shaping policy and operational decisions. Robust evidence of this kind is essential for designing proportionate interventions that protect consumers without imposing unnecessary burdens on operators.

Beyond policy applications, Open Banking data offers scope for methodological innovation. It opens several new avenues for gambling research. For example, the mixed-methods approaches already pioneered with Open Banking protocols (Zendle and Newall, 2024) could be extended to integrate other forms of evidence, including qualitative data. Any study collecting consent could also seek permission to link banking data. Furthermore, these inherently longitudinal datasets allow analysis of behaviour and harm progression over time, with the possibility of designing studies that investigate causal relationships, either by embedding Open Banking data within a randomised controlled trial or through quasi-experimental methods such as regression discontinuity designs.

Ghaharian et al. (2025) is an important and timely contribution to the gambling research literature. It demonstrates the value of large-scale, objective financial data for understanding gambling behaviour, while highlighting areas where further work is needed to maximise the potential of such sources. The study creates opportunities for both researchers and policymakers. Continued research of this kind is essential, not only to refine methodological approaches, but also to strengthen the evidence base that underpins efforts to reduce gambling-related harm across the population, through both academic advancement and informed policy development. As gambling markets evolve, the academic community has a critical role in ensuring that evidence keeps pace with industry innovation, safeguarding public health in an increasingly digital economy.
